# Vanadium(V) oxide arsenate(V), VOAsO_4_

**DOI:** 10.1107/S1600536811004053

**Published:** 2011-02-05

**Authors:** Safa Ezzine Yahmed, Mohamed Faouzi Zid, Ahmed Driss

**Affiliations:** a Laboratoire de Matériaux et Cristallochimie, Faculté des Sciences, Université de Tunis-ElManar, 2092 El-Manar, Tunis, Tunisia

## Abstract

The vanadyl arsenate, VOAsO_4_, has been isolated by a solid-state reaction. The structure consists of distorted VO_6_ octa­hedra and AsO_4_ tetra­hedra sharing corners to build up VAsO_7_ layers parallel to *ac* linked by edge-sharing of VO_6_ octa­hedra, forming a three-dimensional framework.

## Related literature

For the preparation, see: Ezzine *et al.* (2009 ▶). For structural relationships, see: Leclaire *et al.* (2002 ▶); Lii *et al.* (1990 ▶); Haddad *et al.* (1992 ▶); Haddad & Jouini (1994 ▶); Borel *et al.* (1997 ▶). For properties of related compounds, see: Aranda *et al.* (1992 ▶); Daidouh *et al.* (1997 ▶); Nguyen & Sleight (1996 ▶). For bond-valence data, see: Brown & Altermatt (1985 ▶). For related structures with formula *M*O*X*O_4_ (*M* = V, Nb, Mo, Sb; *X* = P, S), see: Amos *et al.* (1998 ▶); Boghosian *et al.* (1995 ▶); Kierkegaard & Longo (1970 ▶); Piffard *et al.* (1986 ▶); Tachez *et al.* (1981 ▶).

## Experimental

### 

#### Crystal data

VOAsO_4_
*M* *_r_* = 205.86
Monoclinic, 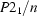

*a* = 6.3338 (7) Å
*b* = 8.2826 (8) Å
*c* = 6.3599 (7) Å
β = 90.19 (1)°
*V* = 333.64 (6) Å^3^
*Z* = 4
Mo *K*α radiation
μ = 12.69 mm^−1^
*T* = 298 K
0.21 × 0.11 × 0.10 mm

#### Data collection

Enraf–Nonius CAD-4 diffractometer
Absorption correction: ψ scan (North *et al.*, 1968 ▶) *T*_min_ = 0.201, *T*_max_ = 0.276
1646 measured reflections
724 independent reflections
672 reflections with *I* > 2σ(*I*)
*R*_int_ = 0.022
2 standard reflections every 120 min  intensity decay: 1.2%

#### Refinement

*R*[*F* ^2^ > 2σ(*F* ^2^)] = 0.018
*wR*(*F* ^2^) = 0.057
*S* = 1.16
724 reflections
65 parameters
Δρ_max_ = 0.63 e Å^−3^
Δρ_min_ = −0.72 e Å^−3^

### 

Data collection: *CAD-4 EXPRESS* (Duisenberg, 1992 ▶; Macíček & Yordanov, 1992 ▶); cell refinement: *CAD-4 EXPRESS*; data reduction: *XCAD4* (Harms & Wocadlo, 1995 ▶); program(s) used to solve structure: *SHELXS97* (Sheldrick, 2008 ▶); program(s) used to refine structure: *SHELXL97* (Sheldrick, 2008 ▶); molecular graphics: *DIAMOND* (Brandenburg, 1998 ▶); software used to prepare material for publication: *WinGX* (Farrugia, 1999 ▶).

## Supplementary Material

Crystal structure: contains datablocks I, global. DOI: 10.1107/S1600536811004053/ru2001sup1.cif

Structure factors: contains datablocks I. DOI: 10.1107/S1600536811004053/ru2001Isup2.hkl

Additional supplementary materials:  crystallographic information; 3D view; checkCIF report

## Figures and Tables

**Fig. 1.**
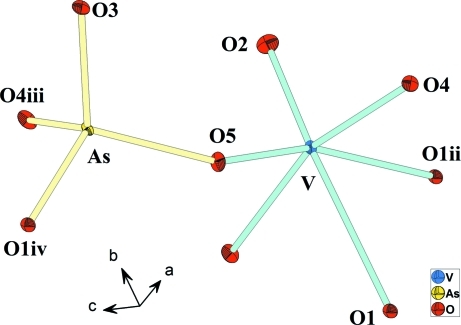
Unité asymétrique dans VOAsO4. Les éllipsoïdes ont été définis avec 50% de probabilité. [codes de symmétrie: (i) x - 1/2, -y + 1/2, z - 1/2; (ii) x - 1, y, z; (iii) x - 1, y, z - 1; (iv) -x + 1, -y, -z + 1; (v) x - 1/2, -y + 1/2, z + 1/2].

**Fig. 2.**
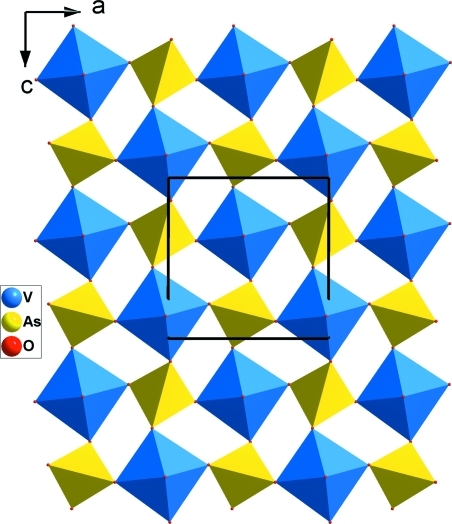
Projection d'une couche VAsO7 selon b montrant la connexion des chaînes infinies VAsO8.

**Fig. 3.**
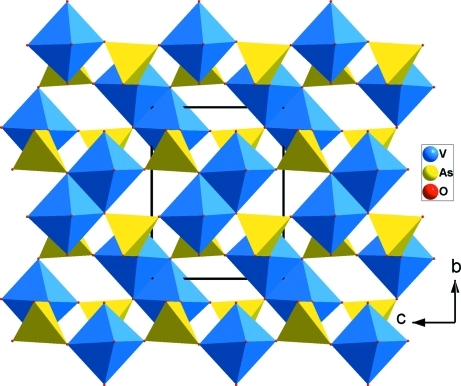
Projection de la structure de VOAsO4 selon a.

**Fig. 4.**
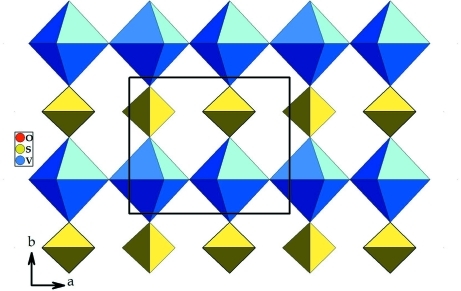
Projection d'une couche VSO7 selon c dans l'oxyde VOSO4.

## supplementary crystallographic information

### Comment

Le vanadium peut adopter différentes coordinations et divers états d'oxydation. En outre, la jonction des polyèdres VO_n_ avec des tetraèdres XO_4_ (X= P ou As), peut mener à des composés possédant des charpentes anioniques ouvertes mixtes uni, bi ou tridimensionnelles (Leclaire *et al.*, 2002; Lii *et al.*, 1990; Haddad *et al.*, 1994; Borel *et al.*, 1997; Ezzine *et al.*, 2009), pouvant manifester certaines propriétés physiques intéressantes notamment: de conduction ionique (Daidouh *et al.*, 1997), d'échange d'ions (Aranda *et al.*, 1992) ou parfois catalytique (Nguyen & Sleight, 1996). L'unité asymétrique dans la structure renferme un tétraèdre AsO_4_ et un octaèdre VO_6_ reliés par mise en commun d'un sommet formant l'unité classique VAsO_9_ (Fig. 1), présentant une distance courte caractéristique d'un groupement vanadyl ( d(V–O)= 1,570 (3) Å). Ces unités se connectent pour établir des chaînes infinies VAsO_8_ parallèles respectivement à a et c. L'association de celles-ci, assurée par partage de sommets entre les polyèdres de nature différente conduit à des couches infinies VAsO_7_ disposées parallèlement au plan ac (Fig. 2). La jonction de ces dernières est réalisée par partage d'arêtes ente octaèdres VO_6_ appartenant à deux couches adjacentes pour conduire à une structure tridimentionnelle (Fig. 3).

La formule empirique de Brown (Brown & Altermatt, 1985) a été utilisée pour le calcul des différentes valences des liaisons qui vérifient bien les valeurs des charges des ions V(5,036) et As(4,936) dans cet oxyde.

La comparaison de la structure de VOAsO_4_ avec des travaux antérieurs de formulation analogue MOXO_4_ (avec M= V, Nb, Mo ou Sb; X= P ou S) révèle la présence des chaînes classiques MXO_8_ dans les composés MOPO_4_ (M= V, Mo, Nb, Sb) ( Amos *et al.*, 1998; Piffard *et al.*, 1986; Tachez *et al.*, 1981; Kierkegaard *et al.*, 1970 ) et VOSO_4_ (Boghosian  *et al.*, 1995) analogues à celles rencontrées dans la phase étudiée. La jonction entre ces chaînes, dans VOPO_4_, MoOPO_4_, NbOPO_4_ conduit aux mêmes types de couches MXO_7_ rencontrées dans notre oxyde VOAsO_4_. Cependant dans les composés VOSO_4_ et SbOPO_4_ ces chaînes se lient  par partage de sommets moyennant les octaèdres MO_6_ et établissent des couches infinies MXO_7_ (Fig. 4). Ces dernières se connectent entre elles par partage de sommets entre les octaèdres MO_6_ dans les phosphates de  niobium, vanadium ou molybdène et par ponts mixtes M-O-X dans le sulfate de vanadium et le phosphate d'antimoine. L'association des couches conduit dans chaque cas à une structure tridimensionnelle.

### Experimental

Des cristaux de la phase VOAsO_4_ ont été obtenus au cours de l'exploration du système Na_2_O–V_2_O_5_–As_2_O_5_. En effet, un mélange réalisé dans les conditions stoechiométriques (1:2:2) à partir des réactifs solides NH_4_H_2_AsO_4_ (préparé au laboratoire, ASTM 01–775), NH_4_VO_3_ (Riedel-De Haën) et NaNO_3_ (Fluka) est finement broyé et pré-chauffé à 573 K pendant une nuit. La température est ensuite portée à 873 K pendant deux jours. Après refroidissement, l'observation du mélange révèle la présence de deux types de cristaux. La phase majoritaire, sous forme parallélepipédique de couleur verte, s'avère moyennant la diffraction des rayons-*X*, le composé NaVAsO_5_ (Haddad *et al.*1992). Un cristal de la phase minoritaire a été donc choisi pour la détermination de sa structure.

### Refinement

Le facteur de consistance interne *R*_int_ calculé pour les facteurs de structure supposés équivalents soit dans les cas orthorhombic ou tetragonal conduit à des valeurs supérieures à 26%. *L*'affinement final est par conséquent mené dans le sysème monoclinic avec une valeur de *R*_int_ égale à 2,2%. Les densités d'électrons maximum et minimum restants dans la Fourier-différence sont situées respectivements à 0,73 Å de O2 et à 0,93 Å de As.

### Crystal data

**Table:**

VOAsO_4_	*F*(000) = 384
*M**_r_* = 205.86	*D*_x_ = 4.098 Mg m^−^^3^
Monoclinic, *P*2_1_/*n*	Mo *K*α radiation, λ = 0.71073 Å
Hall symbol: -P 2yn	Cell parameters from 25 reflections
*a* = 6.3338 (7) Å	θ = 10–15°
*b* = 8.2826 (8) Å	µ = 12.69 mm^−^^1^
*c* = 6.3599 (7) Å	*T* = 298 K
β = 90.19 (1)°	Prism, orange
*V* = 333.64 (6) Å^3^	0.21 × 0.11 × 0.10 mm
*Z* = 4	

### Data collection

**Table:**

Enraf–Nonius CAD-4 diffractometer	672 reflections with *I* > 2σ(*I*)
Radiation source: fine-focus sealed tube	*R*_int_ = 0.022
graphite	θ_max_ = 27.0°, θ_min_ = 4.0°
ω/2θ scans	*h* = −8→8
Absorption correction: ψ scan (North *et al.*, 1968)	*k* = −1→10
*T*_min_ = 0.201, *T*_max_ = 0.276	*l* = −8→8
1646 measured reflections	2 standard reflections every 120 min
724 independent reflections	intensity decay: 1.2%

### Refinement

**Table:**

Refinement on *F*^2^	Primary atom site location: structure-invariant direct methods
Least-squares matrix: full	Secondary atom site location: difference Fourier map
*R*[*F*^2^ > 2σ(*F*^2^)] = 0.018	*w* = 1/[σ^2^(*F*_o_^2^) + (0.0302*P*)^2^ + 0.2701*P*] where *P* = (*F*_o_^2^ + 2*F*_c_^2^)/3
*wR*(*F*^2^) = 0.057	(Δ/σ)_max_ < 0.001
*S* = 1.16	Δρ_max_ = 0.63 e Å^−^^3^
724 reflections	Δρ_min_ = −0.72 e Å^−^^3^
65 parameters	Extinction correction: *SHELXL97* (Sheldrick, 2008), Fc^*^=kFc[1+0.001xFc^2^λ^3^/sin(2θ)]^-1/4^
0 restraints	Extinction coefficient: 0.0051 (10)

### Special details

**Table:**

Geometry. All e.s.d.'s (except the e.s.d. in the dihedral angle between two l.s. planes) are estimated using the full covariance matrix. The cell e.s.d.'s are taken into account individually in the estimation of e.s.d.'s in distances, angles and torsion angles; correlations between e.s.d.'s in cell parameters are only used when they are defined by crystal symmetry. An approximate (isotropic) treatment of cell e.s.d.'s is used for estimating e.s.d.'s involving l.s. planes.
Refinement. Refinement of *F*^2^ against ALL reflections. The weighted *R*-factor *wR* and goodness of fit *S* are based on *F*^2^, conventional *R*-factors *R* are based on *F*, with *F* set to zero for negative *F*^2^. The threshold expression of *F*^2^ > σ(*F*^2^) is used only for calculating *R*-factors(gt) *etc*. and is not relevant to the choice of reflections for refinement. *R*-factors based on *F*^2^ are statistically about twice as large as those based on *F*, and *R*- factors based on ALL data will be even larger.

### Fractional atomic coordinates and isotropic or equivalent isotropic displacement parameters (Å^2^)

**Table:**

	*x*	*y*	*z*	*U*_iso_*/*U*_eq_	
V	0.03529 (6)	0.17105 (6)	0.15935 (6)	0.00436 (16)	
As	0.03636 (4)	0.24811 (3)	0.65771 (4)	0.00357 (14)	
O1	0.0152 (3)	−0.1177 (2)	0.1408 (3)	0.0055 (4)	
O2	0.0313 (3)	0.3600 (3)	0.1395 (3)	0.0113 (5)	
O3	0.2446 (3)	0.3664 (2)	0.7158 (3)	0.0073 (4)	
O4	0.3279 (3)	0.1316 (2)	0.1083 (3)	0.0076 (4)	
O5	0.0968 (3)	0.1379 (2)	0.4438 (3)	0.0071 (4)	

### Atomic displacement parameters (Å^2^)

**Table:**

	*U*^11^	*U*^22^	*U*^33^	*U*^12^	*U*^13^	*U*^23^
V	0.0029 (2)	0.0063 (3)	0.0039 (3)	−0.00016 (15)	−0.00034 (17)	−0.00090 (15)
As	0.00167 (19)	0.0062 (2)	0.0028 (2)	−0.00011 (8)	−0.00033 (12)	0.00060 (8)
O1	0.0066 (9)	0.0064 (9)	0.0035 (8)	−0.0010 (7)	−0.0004 (6)	0.0009 (6)
O2	0.0127 (11)	0.0088 (11)	0.0125 (10)	−0.0003 (7)	−0.0011 (8)	0.0000 (7)
O3	0.0028 (8)	0.0095 (10)	0.0095 (8)	−0.0022 (7)	−0.0001 (7)	−0.0010 (7)
O4	0.0023 (8)	0.0109 (10)	0.0094 (8)	−0.0019 (7)	−0.0004 (6)	−0.0030 (7)
O5	0.0074 (9)	0.0110 (9)	0.0030 (8)	0.0004 (7)	−0.0017 (7)	−0.0006 (7)

### Geometric parameters (Å, °)

**Table:**

V—O2	1.570 (3)	V—O1	2.398 (2)
V—O5	1.869 (2)	As—O3	1.683 (2)
V—O3^i^	1.903 (2)	As—O4^iii^	1.683 (2)
V—O4	1.911 (2)	As—O5	1.683 (2)
V—O1^ii^	1.985 (2)	As—O1^iv^	1.708 (2)
			
O2—V—O5	103.14 (9)	O5—V—O1	84.95 (7)
O2—V—O3^i^	99.33 (9)	O3^i^—V—O1	78.20 (7)
O5—V—O3^i^	89.55 (8)	O4—V—O1	82.66 (7)
O2—V—O4	99.93 (9)	O1^ii^—V—O1	73.88 (8)
O5—V—O4	86.58 (8)	O3—As—O4^iii^	108.07 (10)
O3^i^—V—O4	160.74 (9)	O3—As—O5	108.23 (9)
O2—V—O1^ii^	98.17 (9)	O4^iii^—As—O5	110.52 (9)
O5—V—O1^ii^	158.54 (9)	O3—As—O1^iv^	110.81 (9)
O3^i^—V—O1^ii^	89.55 (8)	O4^iii^—As—O1^iv^	111.25 (9)
O4—V—O1^ii^	87.24 (7)	O5—As—O1^iv^	107.93 (9)
O2—V—O1	171.60 (8)		

Symmetry codes: (i) *x*−1/2, −*y*+1/2, *z*−1/2; (ii) −*x*, −*y*, −*z*; (iii) *x*−1/2, −*y*+1/2, *z*+1/2; (iv) −*x*, −*y*, −*z*+1.
